# The association between lifestyle factors and the composition of the vaginal microbiota: a review

**DOI:** 10.1007/s10096-024-04915-7

**Published:** 2024-08-03

**Authors:** Madjid Morsli, Elise Gimenez, Chloé Magnan, Florian Salipante, Stéphanie Huberlant, Vincent Letouzey, Jean-Philippe Lavigne

**Affiliations:** 1 VBIC, INSERM U1047, Department of Microbiology and Hospital Hygiene, University of Montpellier, CHU Nîmes, Nîmes, France; 2 Department of Gynecology and Obstetrics, University of Montpellier, CHU Nîmes, Nîmes, France; 3 Service de Biostatistique, Epidémiologie, Santé Publique Innovation et Méthodologie, University of Montpellier, CHU Nîmes, Nîmes, France

**Keywords:** Alcohol consumption, Hormonal contraception, Personal hygiene, Psychosocial stress, Smoking, Vaginal microbiota

## Abstract

**Purpose:**

The vaginal microbiota offers valuable insights into women’s sexual health and the risk of developing sexually transmitted infections (STIs) and bacterial vaginosis. Despite the public health implications of changes in the vaginal environment, existing data on this topic remain sparse.

**Methods:**

Following the PRISMA statement guidelines, we consulted five bibliographic databases, focusing on five main daily habits and behaviors. We included only studies published up to October 2023, investigating the influence of personal hygiene, sexual behaviors, hormonal contraception, smoking, alcohol consumption, and psychosocial stress on the vaginal microbiota using next-generation sequencing.

**Results:**

Based on our inclusion criteria, we incorporated 37 studies into this review. Hormonal contraception and personal hygiene were found to promote eubiosis of the vaginal microbiota. In contrast, sexual behaviors, smoking, alcohol consumption, and psychosocial stress were associated with an increased susceptibility to bacterial vaginosis, STIs, and severe pelvic inflammatory diseases due to a modified vaginal microbiota. Black ethnicity emerged as a confounding factor, with this population showing unstable vaginal microbiota. Oral contraception and a stable male sexual partner were found to favor *Lactobacillus* colonization, acting as a protective factor. Conversely, non-hormonal contraception and unprotected or non-penile/vaginal sexual activity increased the incidence of vaginal inflammation and bacterial vaginosis by disturbing the vaginal microbiota and reducing *Lactobacillus* abundance.

**Conclusion:**

Daily habits and lifestyle can influence the composition of the vaginal microbiota, thereby affecting vaginal health. Disturbances in the vaginal microbiota could be associated factors for STIs and vaginosis. Therefore, prioritizing more appropriate management of the vaginal microbiota is crucial.

## Introduction

The microbiota is a rich and complex ecosystem that has been extensively investigated in recent years due to advances in molecular tools [1, 2]. These microbiotas exhibit variation in their composition based on their localization. Among them, the vaginal ecosystem plays a crucial role in women’s health. The formation and evolution of the vaginal microbiome begin at birth, with its composition being influenced by several factors, including genetic, dietary, and environmental factors [3].

In healthy women of reproductive age, the cervicovaginal microbiota is predominantly colonized by *Lactobacillus* spp. Five types of vaginal bacterial communities, termed community state types (CST), have been identified in women of childbearing age [4]. It is common for women to experience rapid changes from one CST to another. Most CSTs are dominated by a *Lactobacillus* species: CST type I (dominated by *Lactobacillus crispatus*), CST type II (*Lactobacillus gasseri*), CST type III (*Lactobacillus iners*), CST type IV (dominated by various other bacteria such as anaerobes), and CST type V (*Lactobacillus jensenii*). CST type IV is further subdivided into subtypes IVA (dominated by BVAB1), IVB (*Gardnerella vaginalis*), IVC0 (*Prevotella*), IVC1 (*Streptococcus*), IVC2 (*Enterococcus*), IVC3 (*Bifidobacterium*) and, IVC4 (*Staphylococcus*) [5].

This ensemble of microorganisms ensures homeostasis and provides a certain degree of protection against pathogenic and/or opportunistic bacteria, parasites, viruses, and certain environmental factors that are harmful to the mucosa. The abundance of *Lactobacillus* within the vaginal microbiota actively contributes to this homeostasis. The acidity of the vaginal pH, fluctuating between 3.8 and 4.5, is an essential element of this protection [3]. Maintaining this natural acidity is crucial as it inhibits the growth of pathogens that struggle to reproduce in this acidic environment. To ensure this acidity, estrogenic impregnation of the vagina promotes the proliferation of vaginal epithelial cells by increasing glycogen deposits. This glycogen serves as a primary source of nourishment for *Lactobacillus*, which uses it to produce lactic acid, thereby contributing to vaginal acidity and positively influencing the colonization of the mucosa by this bacteria genus [6, 7]. Other biological mechanisms, such as competition for nutrient acquisition or modifications of cell adhesion, also protect the lower genital tract [8–10]. Thus, *Lactobacillus* constitute a first line of defense against all vaginal pathogenic microorganisms. They also protect the vaginal epithelium by preventing the attachment of microorganisms to the vaginal wall through the production of various products such as antimicrobial agents (such as bacteriocins, hydrogen peroxide) and biosurfactants [8, 9]. Depending on the bacterial genera/species present and their activity, the vaginal microbiota can trigger various metabolic and immunological responses.

Dysbiosis is the primary factor disrupting the balance of the human vaginal microecology. A non-optimal vaginal microbiota fosters the colonization of pathogenic microorganisms on the vaginal mucosa. This can lead to asymptomatic bacterial vaginosis [11], trigger inappropriate inflammatory responses, and/or provoke abnormal immune responses. This contributes to the appearance of a wide range of pathologies, including severe pelvic inflammatory diseases [5, 9, 12, 13]. Moreover, the presence of non-optimal microbiota has been correlated with increased risks of preterm birth and of contracting sexually transmitted infections (STIs) (e.g., HIV or *Chlamydia trachomatis*), which affect embryonic implantation and increase the risk of miscarriage [1, 3, 5, 14, 15].

The balance within the vaginal microbiota remains relatively fragile, and numerous factors can contribute to its imbalance. In this review, we evaluate the influence of major lifestyle parameters (personal hygiene, use of hormonal contraception, sexual behaviors, psychosocial stress, and tobacco or alcohol consumption) on the evolution and modification of the vaginal microbiota.

## Methods

### Study design

This review aims to understand the influence of daily-life hygiene habits on vaginal health by analyzing the diversity of vaginal microbiota. We reviewed papers that studied vaginal microbiome diversity using metagenomic next-generation sequencing (mNGS). The analysis was based on five main daily-life habits and behaviors of women: personal hygiene, hormonal contraception, sexual activity, psychosocial stress, and smoking and alcohol use.

### Search strategy and selection criteria

The literature search adhered to the Preferred Reporting Items For Systematic Review and Meta-analyses (PRISMA) statement guidelines [16]. We conducted a comprehensive literature search on various databases, including PubMed (NLM database), MEDLINE (OvidSP), Web of Science (Thomson Reuters), Google scholar, Microsoft Academic, Crossref and Semantic Scholar, using Harzing Publish or Perish software (Version 8.1). There were no restrictions regarding language, study status, date of publication, or country, using a MeSH terms string chain. Duplicated studies were removed during the initial screening, and the remaining articles were screened for eligibility based on title and abstract reading. Final inclusion-based screening was performed after full-text reading according to the inclusion criteria utilizing the following keywords: (“Vaginal microbiome” OR “Vaginal microbiota”) AND (“Sexual behavior” OR “Sexual risk behavior” OR “Women who have sex with women” OR “Stable partner” OR “Condom use” OR “Sexual practices”) AND (“Intimate hygiene” OR “Feminine hygiene products” OR “Personal hygiene” OR “Vaginal douching”) AND (“Hormonal contraceptive” OR “Contraceptive pill” OR “Hormonal IUD” OR “Levonorgestrel IUD” OR “Levonorgestrel implant”) AND (“Smoking” OR “Smoking women” OR “Cigarettes” OR “Tobacco” OR “Alcohol consumption”) AND (“Stress” OR “Social induced stress” OR “Psycho-social stress”). The literature search spanned an 11-year period, including articles published between January 1st, 2011, and November 1st, 2023.

**Fig. 1:**
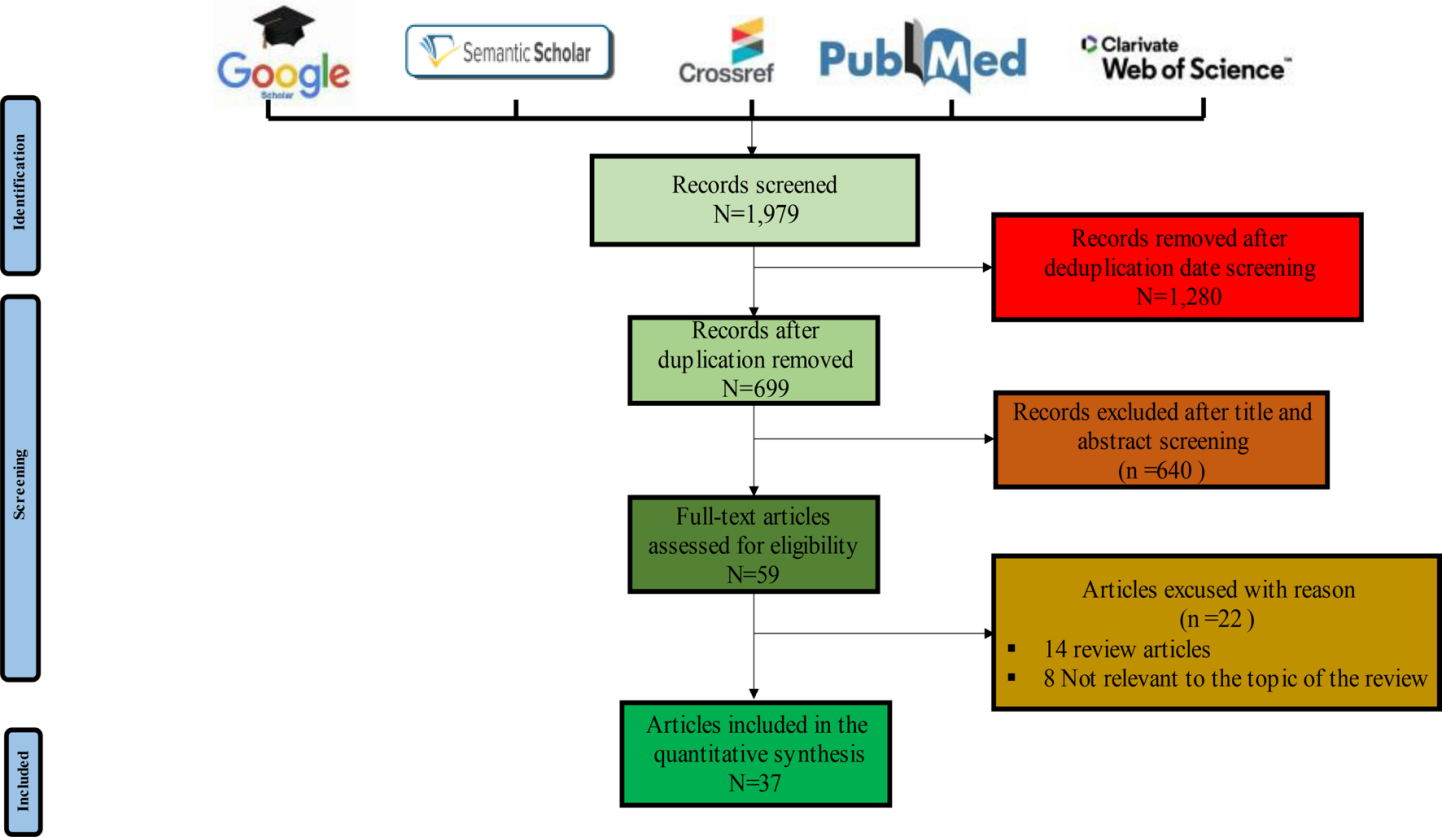
Prisma flowchart screening of eligible articles included in this review. Five bibliographic databases were searched using keywords. After deduplication and screening, 37 articles met the inclusion criteria and were finally included with eligibility in this review

Article screening and data collection were independently conducted by MM, EG and JPL, according to the inclusion criteria. Disagreements were solved through discussion with the four independent reviewers (CM, FS, SH, and VL), as illustrated in Fig. 1.

### Data collection and screening

Only original papers studying the women’s vaginal microbiome were eligible for inclusion in this review. Non-clinical studies, studies on animals, in vitro model studies, and review articles were excluded. As the diversity of the vaginal microbiota may be affected by antimicrobial treatments such as antibiotics and antiviral drugs in the case of HIV infection, studies focusing on women undergoing antibiotic treatments or antiretroviral treatments for HIV, and post-menopausal women were also excluded. Furthermore, studies involving probiotic or live biotherapeutic treatments were also excluded, given their potential influence on the composition of the vaginal microbiota. Due to the focus of our review on the characterization of the vaginal microbiota, only studies using 16S rRNA or shotgun mNGS investigation were included. Confounder bias was limited by studying reproductive women aged 16–45 years old. In this study, we focused on cisgender women assigned female at birth; papers studying transgender women and men in the process of transitioning to female, including hormonal context, were excluded. As we focused on vaginal microbiota characterization, only studies using mNGS strategies were selected, while other molecular or microbiological strategies focusing on Nugent score, cytokines measurement, and other non-genomic investigation were excluded.

Data extracted from the selected studies included: first author name and year of publication, authors’ country, population size, patient age, lifestyle, smoking-alcohol consumption, trial design, methods, objectives, clinical setting, sexual habits and activity, partner sex, marital status, hormonal contraception, personal and menstrual hygiene, outcomes, and microbiology data. The study authors were contacted for additional data if necessary. The data extracted by MM and EG were reviewed by CM and FS and validated by SH, VL and JPL.

### Role of the funding source

No funding was received for this study, and no commercial or financial relationships have been established as a potential conflict of interest.

## Results

### Study inclusion

A literature search on PubMed, Google Scholar, Semantic scholar, Crossref, and Web of Science databases yielded 1,979 articles. After deduplication, 1,280 studies were removed. Further screening based on title and abstract reading eliminated 640 articles. Full-text screening of the remaining 59 articles excluded 22 studies (14 reviews and 8 papers not relevant to the review’s topic). Ultimately, 37 studies were definitively selected for this review (Fig. 1; Table 1).

**Table 1:** Study characteristics and outcomes investigated

Reference	Country	Participant ethnic background	Population size	Sample category	Smoking-alcohol consumption	Psychological stress	Hormonal contraception at admission	Personal hygiene	Sexual activity
[16]	Kenya	African	58	Vaginal swab	No	NI	No	Yes	Yes
[17]	Netherlands	Dutch-Caucasian (*n* = 22), Mixed origin (*n* = 3)	25	Vaginal swab	mai-25	NI	15/25	Yes	Sex with men only (*n* = 24), Sex with women only (*n* = 1)
[18]	USA	Caucasian (*n* = 97), Black (*n* = 104), Asian (*n* = 96), Hispanic (*n* = 97)	394	Vaginal swab	NI	NI	108	67	Yes (Sex toys use = 59)
[20]	USA	NI	26	Vaginal swab	No	No	Yes	NI	NI
[21]	Argentina	Hispanic	101	Vaginal swab	No	NI	Yes	NI	Heterosexual relationships
[22]	South Africa	Mixed origin	130	Vaginal swab	No	No	Yes	45/130	119/130 (with multiple partners = 12)
[23]	USA	Caucasian	13	Cervical and vaginal swab	No	No	Yes	NI	NI
[24]	USA	Black (*n* = 292), Asian (*n* = 11), Caucasian (*n* = 234), Hispanic (*n* = 40), Other (*n* = 27)	682	Culture Swab	NI	NI	670	66	654 (226 > 2 partners)
[25]	USA	Mixed origin (*n* = 38), Black (*n* = 15)	53	Vaginal swab	mars-53	No	No	NI	Yes (with multiple partners *n* = 43)
[26]	Canada	Black	58	Vaginal swab	NI	NI	36	23	Yes (Sex workers)
[27]	USA	Hispanic White (*n* = 9), Black (*n* = 16)	25	Mid-vaginal swab	No	NI	Yes	NI	No practice 2 days before
[28]	USA	Black (*n* = 23), White (*n* = 48), Other (*n* = 5)	76	Vaginal swab	NI	NI	NI	Yes	Yes
[29]	Australia	Caucasian (*n* = 29), Central & South-East Asian (*n* = 19) Indian/Sri-Lankan (*n* = 4)	52	Vaginal swab	2	NI	14	5	33 (non-coital sexual activities only *n* = 19)
[30]	USA	African-American (*n* = 15), Asian/Pacific islander (*n* = 4), Hispanic (*n* = 3), Mixed race (*n* = 4), White (*n* = 14)	40	Mid-vaginal swab	20	NI	32	9	Yes
[32]	Netherlands	Dutch (*n* = 117), African Surinamese (*n* = 116), S-Asian Surinamese (*n* = 103), Turkish (*n* = 89), Moroccan (*n* = 103), Ghanaian (*n* = 82)	610	Vaginal swab	569	NI	241	146	Yes (with female partner *n* = 26)
[33]	USA	White (*n* = 14), Black (*n* = 16), Hispanic (*n* = 1), Other (*n* = 2)	33	mid-vaginal swabs	NI	NI	Contraceptive pill (*n* = 7), Tubal ligation (*n* = 15)	33	Yes
[34]	USA	White (*n* = 291), Black (*n* = 6), American-Indian (*n* = 1), Asian (*n* = 5), Hispanic (*n* = 1)	308	Vaginal swab	200	NI	275	NI	> two partners (*n* = 92)
[35]	USA	White (*n* = 62), Black (*n* = 1), Asian (*n* = 20), Hispanic (*n* = 3), Other (*n* = 10)	97	Vaginal swab	14	NI	40	No	71
[36]	USA	NI	62	Cervicovaginal lavage	43	NI	11	NI	52
[37]	Brazil	Black(*n* = 333), White (*n* = 251), Other (*n* = 25)	609	Vaginal swab	255	NI	143	174	577 (66 > 2 partners)
[38]	USA	NI	39	Mid-vaginal swabs	Yes	NI	No	NI	39 sex with partner
[39]	Brazil	Black (*n* = 229), White (*n* = 193), Other (*n* = 20)	422	Vaginal swab	Alcohol (*n* = 122), Smoking (*n* = 44)	NI	Oral (*n* = 152), Injectable (*n* = 39)	313	420 (36 > 2 sexual partners)
[40]	Canada	NI	67	Vaginal swab	NI	NI	18	NI	Yes (sex workers *n* = 48)
[41]	Netherlands	Dutch (*n* = 80), Surinamese/Dutch Antilles (*n* = 24), Asian (*n* = 6), Turkish (*n* = 2)	122	Vaginal swab	NI	NI	NI	NI	Yes
[42]	USA	African-American	79	Vaginal swab	NI	NI	NI	31	Yes (43 ≥ 2 partners)
[44]	Australia	Australia/New Zealand (*n* = 33), Other (*n* = 42)	75	Vaginal swab	46	NI	19	26	Yes (sex workers *n* = 5)
[45]	Australia	White Australian (*n* = 86), Other (*n* = 14)	100	Vaginal swab	53	NI	NI	20	Yes (WSW)
[46]	USA	Black	21	Vaginal swab	No	Yes	No	Yes	Yes, sex with partner
[47]	Japan	Japanese	54	Nylon flocked ESwab	NI	NI	NI	NI	Yes (Sex workers *n* = 8)
[48]	Canada	African	168	Vaginal swab	NI	NI	Yes	NI	Yes (sex workers *n* = 102)
[49]	USA	Hispanic (*n* = 58) Asian (*n* = 71), White (*n* = 78) African-American (*n* = 78)	285	Vaginal swab	NI	NI	NI	43	222 (with multiple partners *n* = 23)
[50]	USA	American-Indian	70	Vaginal swab	26	Yes	No	18	68
[51]	USA	Black (*n* = 435), White (*n* = 137)	572	Vaginal swab	NI	Yes	No	NI	423 (with multiple partners *n* = 34)
[52]	USA	African-American (*n* = 1,228), Caucasian (*n* = 416)	1,664	Vaginal swab	480	NI	No	193	496
[53]	USA	Asian/Pacific Islander (*n* = 4), White (*n* = 12), African-American (*n* = 14), Hispanic (*n* = 3), Mixed race (*n* = 3)	36	Mid-vaginal swabs	17	NI	No	36	30/36 (with multiple partners *n* = 4)
[54]	USA	African-American	42	Vaginal swab	11	NI	12	No	mixed partner (*n* = 9)
[59]	USA	Black (*n* = 109), Caucasian (*n* = 3)	112	Vaginal swab		NI	NI	NI	NI

Abbreviations: WSM = women having sex with men, WSW = women having sex with women, WSWM = women having sex with women and men, NI = no information

### Classification of the included studies

The 37 studies included in this review originated from various countries: 22 (59.5%) were from the USA, three (8.1%) were from Australia, Canada, and the Netherlands, two (5.4%) were from Brazil, and one each from Argentina, Japan, Kenya, and South Africa. The studies were either retrospective (*n* = 20) or prospective (*n* = 16), with one study not providing this information.

The studies encompassed 7,380 childbearing women, of which 3,641 (47.9%) were of African and African American ethnicity, 2,329 (31.6%) were of White-Caucasian origin (including European, North-African, and Turkish origins), 516 (7%) were of Asian and Pacific Islander origins, 387 (5.2%) were of Hispanic and American Indian origins, 178 (2.4%) were of multiracial origin, and no racial information was provided for the remaining women (*n* = 339 (4.6%)).

### Influence of personal hygiene on vaginal microbiota

Personal hygiene could influence the stability of the vaginal microbiota. In the literature, vaginal douching or other intravaginal practices (IVP) (such as intravaginal cleansing or insertion of products) represented the main studied practices. A wide variety of these intimate hygiene practices are influenced by religion, social and cultural traditions, or patients’ education. Thus, three papers reported the effect of personal hygiene on vaginal microbiota [16–18].

The study by Van Der Veer et al. evaluated the effect of a commercial douche on the vaginal microbiota in 25 healthy Dutch women (median age 24 years (22–29)), of whom 60% used combined oral contraceptives. The authors found that the composition of the vaginal microbiota and vaginal pH were not affected by vaginal douching, while menstruation was reported as an influencing factor involved in modifying the vaginal microbiota, doubling the presence of anaerobes frequently associated with vaginosis (OR = 1.7 with a 95%CI [1.0-2.8]). Similarly, douching during menstruation significantly increased the risk of dysbiosis with an OR of 2.6 times (OR = 2.6 with a 95% CI [1.0-6.5]), compared to patients without menstruation (*p* = 0.099). Moreover, vaginal douching appeared to promote *Candida albicans* infections [18], probably due to a proinflammatory vaginal immune environment.

Another study evaluated the impact of IVP among Kenyan women aged 18 to 45 years at risk of HIV acquisition. No significant difference was observed in the composition of their vaginal microbiota, even three months after IVP cessation. However, both studies are difficult to generalize to the entire population as they were conducted at single center (Netherlands and Kenya) with limited sample sizes (25 and 58 patients, respectively). Additionally, the 3-month follow-up duration might be too short, and the cessation of IVP might have been overestimated [17, 18].

A recent observational study conducted on 33 sexually active women revealed that douching cessation alone was not associated with a modification of the vaginal microbiota or an increased risk of bacterial vaginosis onset. Other extrinsic factors (such as antibiotic use, lubricants, diet, smoking cessation or condom use) would influence the vaginal microbiota much more [19].

### Influence of hormonal contraception on vaginal microbiota

A 2019 United Nations report showed that approximately 407 million women worldwide used hormonal contraception, with 17% using intrauterine devices, 16% using oral contraceptive pills (OCP), 8% using injectable contraceptives, and 2% using an implant [20]. Since the vaginal microbiota is altered by the balance between estrogen and progesterone [21], it was likely that hormonal contraception could also influence changes between CSTs present in the vagina and colonization by *Lactobacillus* [22].

Studies evaluated various contraceptive solutions used systemically or non-systemically, including OCP, an injectable contraceptive and a levonorgestrel intrauterine system (LNG IUS). They revealed that OCP played a significant protective role in the vaginal microbiota [21, 23–25]. Indeed, among women using this method of contraception, the presence of H_2_O_2_ producing-lactobacilli was much more significant, with a vaginal microbiota mainly belonging to CST type I and much lower risks of bacterial vaginosis. Women using depot-medroxyprogesterone acetate (DMPA), an injectable contraceptive, also exhibited a predominance of CSTs containing predominantly lactobacilli, although these bacteria species produced much less H_2_O_2_ compared to what had been observed for OCP [26–28]. In these patients using DMPA, a high presence of *Atopobium vaginae* and/or *Prevotella bivia* was observed, indicating the presence of bacterial vaginosis at the time of sampling. Evaluation of the LNG IUS showed similar results with an increase in bacterial vaginosis and the predominance of CSTs less populated by *Lactobacillus*, thus tending towards a non-optimal vaginal microbiota. These results were obtained from two studies, a large cohort of 682 women, allowing for the control of confounding factors (including birth control, ethnicity, education, employment, health habits, dietary habits, sexual history, and the women age), and a second one with a small sample size (*n* = 11), suggesting an obvious lack of statistical power [24, 25]. However, since these two studies observed similar results, it seems likely that LNG IUS could significantly influence the composition of the vaginal microbiota. Furthermore, in the large cohort conducted by Brooks et al., the use of combined OCP with the intrauterine system favored the presence of H_2_O_2_-producing *Lactobacillus* in these patients compared to women exclusively using the intrauterine system [24].

However, studies comparing LNG UIS to copper intrauterine devices (CIUD) showed that the former device was more commonly associated with an increase in microbiota belonging to CST type I and greater stability of the vaginal microbiota after insertion of the LNG IUS placement compared to CIUD. As a result, no statistical difference was found. This is likely due to the lack of statistical power in the included studies, which can be attributed to the very limited size of the study population [24, 25, 29, 30].

**Fig. 2:**
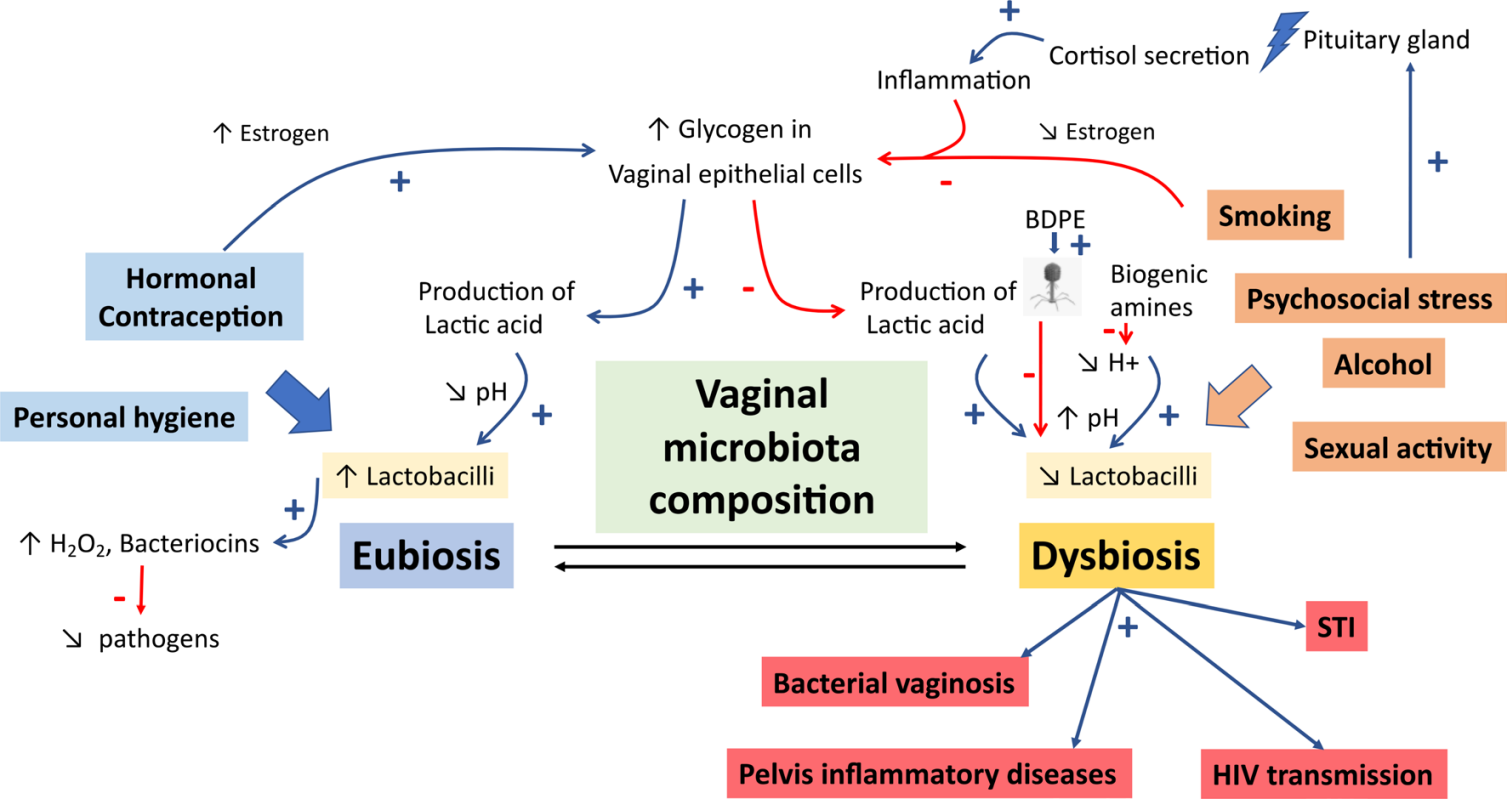
Effects of different lifestyle factors on the vaginal microbiota composition and their consequences. BDPE, benzo[alpha]pyrene diol epoxide; pH, vaginal pH; STI, sexually transmitted infection

In studies examining the influence of lifestyle on the vaginal microbiota, a major bias factor must be systematically considered: the ethnic origin of the patients. Indeed, this factor strongly influences the composition of the vaginal microbiota, especially depending on the hormonal contraception used. As 28.5% of included articles studied African or African American women, almost 50% of participants were of black ethnicity (Table 1). Women of African and African-American origin exhibited a much less stable vaginal microbiota over time, were more prone to STIs, and had higher risks of contracting HIV-1 [31]. Due to the high levels of hormonal contraception in this population, many more studies focused on African/Sub-Saharan women to assess the effectiveness of subcutaneous injectable contraceptives (such as DMPA) in preventing bacterial vaginosis [26–28]. Different studies have shown that a higher proportion of women presented a more diversified vaginal microbiota with the use of DMPA (in Sub-Saharan women according to 12 research sites) [28], without an increase in the occurrence of STIs, as previously suggested [24]. This result could be explained by a hypoestrogenic status induced by DMPA. As noted earlier, estrogens influence glycogen deposition on the vaginal wall, allowing colonization of the vaginal epithelium by *Lactobacillus* producing lactic acid, and thus contributing to maintaining the acidic pH of the vagina (Fig. 2). Conversely, this hypoestrogenic status could be associated with a lack of lactobacilli abundance and greater diversity of the vaginal microbiota, promoting infections [26]. Comparison of DMPA use by ethnic origin (African and African-American vs. Caucasian) among women with identical CSTs at baseline showed that Caucasians maintained CSTs dominated by *Lactobacillus* over time, while women of African and African-American origin had modifications in their microbiota that became more diverse, with a depletion of lactobacilli [32]. These results highlight the association between hormonal balance, physiological factors, and the composition and diversity of the vaginal microbiota. This association appears to be influenced by ethnic origin and the use of the contraceptives, particularly DMPA (Fig. 2).

### Influence of sexual behavior on vaginal microbiota

A potential link between sexual activity and the vaginal microbiota was assessed in 14 articles, establishing that sexual activity can influence the composition and diversity of this microbiota and also plays a major role in lower genital tract inflammation and the occurrence of STIs [19, 33, 34]. Furthermore, certain sexual habits such as penile-vaginal sex, receptive oral sex, the use of sex toys, and condom use could also directly modify the composition of the vaginal microbiota [35].

The relationship between sexual activity and the vaginal microbiota was studied in 97 pubertal virgin patients to assess the evolution of the vaginal microbiome before and after the onset of sexual activity [36]. In this study, women who had not had sexual intercourse by the end of the study follow-up maintained a stable composition of their vaginal microbiota over time, mainly colonized by *Lactobacillus* species. In contrast, the vaginal microbiota of sexually active women belonged to CST type IVB with a predominance of *Gardnerella vaginalis*. *G. vaginalis* is typically found in low abundance in the normal vaginal microbiota and contributes to the protection against genital infections by maintaining the acidity of the vaginal pH. However, a high relative abundance of *G. vaginalis* can have adverse effects on reproductive and sexual health outcomes by increasing the risk of bacterial vaginosis [36]. Similar results were observed in studies examining the effects of sexual intercourse with a stable partner [31, 37, 38]. Indeed, a comparison of vaginal microbiota composition between women with stable partners and those with multiple partners showed no significant difference between the two populations, whereas women engaging in penile-vaginal intercourse were more likely to be colonized by a CST type III, with *G. vaginalis* being present as a minority [30, 38, 39]. These results suggest that this CST type III with a predominance of *L. iners* may not be protective and may represent a risk factor for developing STIs. Furthermore, studies suggest that *G. vaginalis* is dynamically acquired from the penile skin microbiota of a sexual partner. This reinforces the hypothesis that *G. vaginalis* can be sexually transmitted during unprotected intercourse [29, 40]. Finally, van Houdt et al. observed that having sexual activity but living separately from the partner was also associated with a modification of the vaginal microbiota and with an increased risk of infection, particularly with *C. trachomatis* [42].

Studies on the vaginal microbiota of women with or without bacterial vaginosis within monogamous heterosexual couples show that penile vaginal intercourse influences the composition and diversity of the vaginal microbiota, promoting inflammation of the vaginal epithelium [43]. These modifications of the vaginal microbiota occur, again as with *G. vaginalis*, upon contact with the penile skin microbiota of the partner during unprotected intercourse. Similarly, in sex workers, modifications of the vaginal microbiota have been observed, particularly during oro-vaginal intercourse where the presence of the *Shuttleworthia* genus in the vaginal microbiota is associated with an increased risk of bacterial vaginosis in this population [29, 40, 42]. *Shuttleworthia* is a commensal bacterial genus of the oral cavity. Its presence in the vaginal cavity is associated with a significant increase in vaginal microbiota diversity correlated with an absence (or a very significant decrease) of *Lactobacillus*, contributing to favoring the risk of acquiring STIs, especially HIV, among sex workers [40]. It could also be related to human papillomavirus (HPV) infections with low-grade squamous intraepithelial lesions, precancerous lesions of the cervix [44]. Further research is needed to clarify the role and impact of sexual behaviors on these vaginal bacterial communities [40, 45].

Other sexual habits, including homosexual or bisexual activity, receptive oral intercourse, or sharing unwashed sex toys, significantly increase the risk of modifying vaginal bacterial communities towards CST type IV, thus favoring infections [39, 45]. This risk could be reduced by treating both sexual partners [39, 44, 46]. Furthermore, prostitution is a significant risk factor in modifying the vaginal microbiota. It is often associated with diverse sexual intercourse, multiple partners, random condom use, and high contraceptive use [40, 47, 48]. The vaginal microbiota of sex workers is mostly lacking in *Lactobacillus*, with vaginal bacterial communities of type CST IV, leading to a much higher risk of contracting STIs, especially HIV [40, 48]. It is worth noting that studies on sex workers have been conducted mainly in developing countries, creating a likely analysis bias but also highlighting a major public health issue in these countries that would require the implementation of prevention plans to protect sex workers and limit the spread of STIs, particularly HIV.

### Influence of psychosocial stress on vaginal microbiota

The function of the human immune system is influenced by high levels of stress, potentially increasing susceptibility to infections [18]. Although the mechanisms underlying this susceptibility are not clearly established, the vaginal microbiota appears to be altered by perceived stress (Fig. 2). This modification of microbiota could be due to the presence of biogenic amines and pro-inflammatory responses altering vaginal physiology [23]. Biogenic amines are produced by the decarboxylation of amino-acid, which involves the consumption of hydrogen ions. Decreased hydrogen ions would increase vaginal pH, completely destabilizing the balance of the vaginal microbiota, especially that of lactobacilli. This phenomenon would thus favor the vaginal colonization by a more diversified bacteria community in women exposed to psychosocial stress [17, 49]. Furthermore, stress increases adrenal corticotropic hormone released by the pituitary gland. This hormone weakens immune defenses by inducing pro-inflammatory responses, and causing cortisol secretion, which binds to beta-adrenergic receptors on immune cells, reducing their effectiveness. Cytokines, polypeptide mediators of immunity and inflammation, also play an essential role by acting as true neurotransmitters in the central nervous system during stress, affecting the immune response. Moreover, the destruction of epithelial cells during the inflammatory process caused by pH modification would prevent the deposition of vaginal glycogen, limiting the carbon, which is an essential nutrient source used by *Lactobacillus* species in the vaginal microbiota (Fig. 2) [18].

Among American-Indian women living with a high perceived stress level and presenting high rates of STIs, an association between the presence of CST type IV and life traumas and self-esteem issues has been observed, as well as the implication of stressful situations in the association between these CST type IV and bacterial vaginosis [51].

Women with a 5-unit increase in stress, assessed by Cohen’s 10-point Perceived Stress Scale (PSS-10), had a higher risk of developing bacterial vaginosis, associated with a low abundance of *Lactobacillus* and a predominance of anaerobic bacteria [51]. In these women, the risk of transitioning from CST type III to CST type IV increased by 26%. In contrast, only 19% of women with CST IV at baseline had a change in their vaginal CST evolving toward types I or II after a 5-unit increase in the PSS-10 score. This study thus confirmed the association between psychosocial stress and the modification of vaginal microbiota, often resulting in a decrease in *Lactobacillus* (Fig. 2) [16, 26, 51]. Even more notably, psychosocial stress has been demonstrated as a factor influencing the composition of the vaginal microbiota in > 80% of women with African or African-American origin [46, 50, 51]. However, this latter result should be moderated since we have previously seen that in this population, the percentage of women with vaginal bacterial communities of CST III and IV was significantly higher than in other populations regardless of psychosocial stress [46, 52].

### Influence of tobacco and alcohol consumption on vaginal microbiota

Seven studies examined the effect of smoking or alcohol consumption on the vaginal microbiota. The majority of these studies suggested that smoking was a confounding factor and introduced bias into the analyses [34, 36–38]. Only three studies to date have investigated the direct relationship between smoking and the composition of the vaginal microbiota [30, 52, 53]. Thus, smoking was identified as a factor influencing the diversity of the vaginal microbiota, increasing by 25 times the risk for a woman to have a CST type IV bacterial community [31]. This result can be explained by the high levels of biogenic amines present in the vagina, reducing the abundance of *Lactobacillus* species and facilitating colonization by pathogenic bacteria, leading to vaginal odor and increased susceptibility to urogenital infections [30, 53]. The low abundance of *Lactobacillus* in smokers compared to non-smokers suggests that smoking cessation is a good strategy to reduce the risk of developing bacterial vaginosis [30, 52]. In four women who underwent a smoking cessation program with initially high Nugent scores and a CST type IV bacterial community, three of them showed low Nugent scores and a CST dominated by *Lactobacillus* after 12 weeks. The last participant had a low Nugent score, and the composition of her vaginal microbiota was beginning to shift towards a CST type I.

While the majority of studies considered alcohol consumption as an exclusion criterion, only one paper discussed its effect on the vaginal microbiota. Despite the limited number of studies in this area, it was observed that alcohol consumption could potentially influence the vaginal microbiota in a manner similar to tobacco, thereby increasing the risk of bacterial vaginosis [52]. This unique investigation focused on the importance of alcohol in conjuction with other factors such as smoking, body mass index, and sexual activity. However, ethnicity emerged as the most significant factor associated with changes in the vaginal microbiota. Given these findings, the direct role of alcohol consumption in shaping the composition of the vaginal microbiota remains to be clarified [52].

### Limitations of studies

Most of studies conducted to date (58%) were single-center studies, primarily focused on women of African or African-American origin (≈ 50%). This focus has introduced an ethnic bias into the main results. The limited number of studies in each category, the small size of the study populations, and the lack of long-term follow-up have made comparative analysis unfeasible, leaving the influence of lifestyle factors on vaginal microbiota unclear. This was particularly evident in the study by Brotman et al., which evaluated the influence of smoking cessation on the vaginal microbiota in a small sample of only four women [31].

Only three studies have evaluated the association between psychological stress and the vaginal microbiota, and two on the role of personal hygiene [16, 17, 46, 50, 51]. Furthermore, no work has provided a real-time follow-up of the vaginal microbiota. Only two studies followed patients for a period of three months, a duration insufficient to draw definitive conclusions. This suggests that the authors conclusions may overestimate the influence of discontinuing IVP on the composition of the vaginal microbiota [17, 18]. Only one study examined the effect of alcohol consumption on the vaginal microbiota. However, this study was confounded by other factors such as smoking, ethnicity, and sexual partners, which were not the primary focus of the studies. This limits the ability to establish a direct link between alcohol and changes in the vaginal microbiota [52].

Ethnicity was a significant limitation in several studies, with a predominance of African/African-American participants potentially biasing the interpretations. To support the final results, a more balanced population is needed [46, 52]. It’s worth noting that Afro-American women typically have vaginal bacterial communities of CST type III and IV, which are often associated with bacterial vaginosis. Furthermore, this population is characterized by a more unstable vaginal microbiota compared to women of other origins, increasing the risk of the vaginal microbiota evolving into CST type IV.

All the limitations and difficulties in interpreting the currently published studies could be summarized in the paper by Novak et al. [39]. The authors reported that unprotected sexual intercourse, number of sexual partners, and low or non-existent education could be associated with a vaginal microbiota dominated by *L. iners*, which is contrary to many studies’ findings. Additionally, Novak et al. observed that this predominance of lactobacilli did not protect the vaginal microbiota from colonization by *Candida* sp. [39].

## Discussion

The objective of this review was to understand the influence of lifestyle factors on the composition of the vaginal microbiota. Numerous studies have established a link between non-optimal vaginal microbiota, bacterial vaginosis and their consequences. These complications are associated with an increased risk of contracting STIs, particularly HIV, and with promoting preterm births, and pelvic inflammatory diseases [12, 13, 16, 32, 36, 40, 54]. During dysbiosis, an imbalance in the vaginal microbiota occurs, favoring the presence of numerous bacteria, mainly represented by strict anaerobic species. These bacteria disrupt the epithelial barrier of the vaginal mucosa by secreting metabolites and enzymes, altering the pH, and leading to inflammation, thereby promoting bacterial vaginosis (Fig. 2) [55, 56, 57]. For better management of patients with vaginosis, evaluating factors influencing the composition of the vaginal microbiota, particularly those promoting non-optimal microbiota, is an essential step [7, 9, 11, 13, 56]. We found that only 37 studies have analyzed the influence of these factors on the vaginal microbiota, highlighting the vast scope of research in this field (Fig. 1). It is also important to note that primarily studies on bacterial communities have been conducted in the literature. However, advances in the vaginal mycobiome and viral microbiota are burgeoning, and these microorganisms could be of interest in the evolution towards vaginoses [58, 59].

This review highlights the significant role of ethnicity in modulating the vaginal microbiota, with women of African or African-American origin being highly represented. This focus stemmed from the unique characteristics of the vaginal microbiota in this population, with CST type III and type IV status being prevalent, thereby increasing the risk of acquiring STIs and HIV (Table 1) [24, 37, 51, 52, 54]. Ethnicity could also act as a confounding factor, especially among black women who tend to have a more unstable vaginal microbiota compared to other ethnicities, making them more susceptible to transitioning to less optimal vaginal CST [9, 11, 37]. This overrepresentation of women of African or African-American origin could potentially influence the interpretation of study outcomes in other populations [24, 52].

Despite the non-statistical significance of findings in the majority of studies, some lifestyle factors may be associated with maintaining the composition of the vaginal microbiota. In this way, the effects of contraception use on the vaginal microbiota are notably significant [24, 26–28]. However, DMPA and other injectable contraceptives, which were primarily evaluated in the studies, account for only 8% of contraception use worldwide, while OCPs represent 16% [21, 23, 25–27]. The use of OCP and sexual activity have been associated with a predominance of *L. crispatus*, which has been reported as a protective factor in Caucasian women [29]. In contrast, non-hormonal contraception has been linked to an increased incidence of genital inflammation and bacterial vaginosis. DMPA, in particular, is suspected of disrupting the diversity of the vaginal microbiota by reducing the abundance of *Lactobacillus* and decreasing H_2_O_2_ production [21, 24, 27, 29, 54]. Furthermore, unprotected sex and a higher number of sexual partners among women with limited educational levels were associated with a dominance of protective *L. iners*, but without protection against other colonizers such as *Candida* sp. [39]. The vaginal microbiota of sex workers more frequently belonged to CST type IV with a high risk of developing bacterial vaginosis. This may be due to the important number of sexual partners that could influence the vaginal microbiota composition [26, 45, 47, 48, 59, 60]. According to the reviewed studies, sexual behaviors must be managed and treated with more precautions [40, 51]. Finally, stress, smoking and alcohol consumption could influence the vaginal microbiota and increased its diversity [8, 17, 30, 46, 49–52]. Smoking and alcohol consumption may also confound the risk of bacterial vaginosis, rather than directly increasing the risk of STIs or vaginosis by reducing *Lactobacillus* abundance.

In conclusion, this study highlights the involvement of lifestyle habits in vaginal microbiota modification. Communication about these studies and their results must be strongly considered. Efforts to inform women about the benefits of a healthy vaginal microbiome must be made in the future. Reducing dysbiosis and stabilizing the vaginal microbiota could be an essential pathway in the fight against bacterial vaginosis and STIs, which have been on the rise in recent years. The risks of preterm birth or failure of artificial insemination, cervical cancer, HIV transmission, and other STIs are correlated with the composition and stability of the vaginal microbiota. Considering this microbiota as an essential and relevant component of women’s health is a significant public health issue.

## Data Availability

No datasets were generated or analysed during the current study.
